# Comparison of partial nephrectomy and radical nephrectomy for cystic renal cell carcinoma: a SEER-based and retrospective study

**DOI:** 10.1038/s41598-023-34950-x

**Published:** 2023-05-17

**Authors:** Wenhao Lin, Zhenggang Yang, Ling Yan, Jun Dai, Chen Fang, Yining Hao, Danfeng Xu, Jin Zhang, Juping Zhao

**Affiliations:** 1 Department of Urology, Ruijin Hospital, Shanghai Jiao Tong University School of Medicine, Shanghai, China; 2 Department of Urology, Renji Hospital, Shanghai Jiao Tong University School of Medicine, Shanghai, China; 3 Department of Radiology, Ruijin Hospital, Shanghai Jiao Tong University School of Medicine, Shanghai, China

**Keywords:** Cancer, Nephrology, Oncology, Urology

## Abstract

Cystic renal cell carcinoma (cRCC) is uncommon and surgical indication remains controversial. We compared radical nephrectomy (RN) with partial nephrectomy (PN) in patients with cRCC using data from the Surveillance, Epidemiology and End Results (SEER) database and a retrospective cohort including 106 cRCC patients hospitalized in Ruijin and Renji Hospitals from 2013 to 2022. The baseline characteristics between RN and PN groups in both cohorts were adjusted by propensity score-matching (PSM). A total of 640 patients were included in the SEER cohort. Before PSM, PN group in the SEER cohort had a lower level of T stage (*p* < 0.001) and comprised more Caucasians (*p* < 0.001). After PSM, RN was associated with worse overall survival (*p* < 0.001) and cancer-specific survival (*p* = 0.006) in contrast to PN. In the Chinese cohort, 86 patients who underwent PN and 20 patients who underwent RN were finally included. The mean proportions of estimated glomerular filtration rate preserved after RN were worse than PN. Therefore, PN should be preferred in cRCC patients.

## Introduction

Cystic renal cell carcinoma (cRCC) is a term that varies among different studies for decades, accounting for 5–15% of all RCCs according to various definitions^1^. Conventionally, cRCC is considered as a series of malignant renal masses with a more than 75% cystic area according to imaging, mostly with favorable outcomes^2,3^. It is worth noting that tumor necrosis is often identified as a pseudocystic change with poor prognosis, and therefore many urologists and pathologists do not classify it as cRCC.

Before 2016, cRCC used to be roughly as unilocular and multilocular RCC mainly according to radiological features. Yet, more recently, the International Society of Urological Pathology (ISUP) recommended renaming part of multilocular cRCC as a multilocular cystic renal neoplasm of low malignant potential (MCRNLMP), which is entirely composed of numerous cysts with septa containing small groups of clear cells without expansile growth. However, there is no specific pathological term for cRCCs that fails to match the MCRNLMP criteria. Therefore, some authors use predominantly cystic renal cell carcinoma to describe this type of RCC. Also, previous studies have shown that predominantly cRCCs also rarely recur or metastasize^4,5^.

In the past, radical nephrectomy (RN) was frequently applied to cRCC for fear of intraoperative cyst rupture. More recently, partial nephrectomy (PN) has been recommended for small renal tumors because it can better preserve postoperative renal function and is as effective as RN. Therefore, more and more urologists are trying to apply PN in cRCC patients. Several studies have reported PN as an effective and safe surgery for cRCC^6,7^, and cyst rupture seems unconnected with recurrence^8^. However, with PN becoming more prevalent for cRCC, the superiority of PN needs to be further validated in a large cohort in comparison with RN.

The present study aimed to investigate the prognostic significance of RN and PN in patients with cyst-associated RCC based on the Surveillance, Epidemiology, and End Results (SEER) database and patients with predominantly cRCC from a Chinese cohort.

## Results

### Descriptive characteristics of cRCC

**Figure 1:**
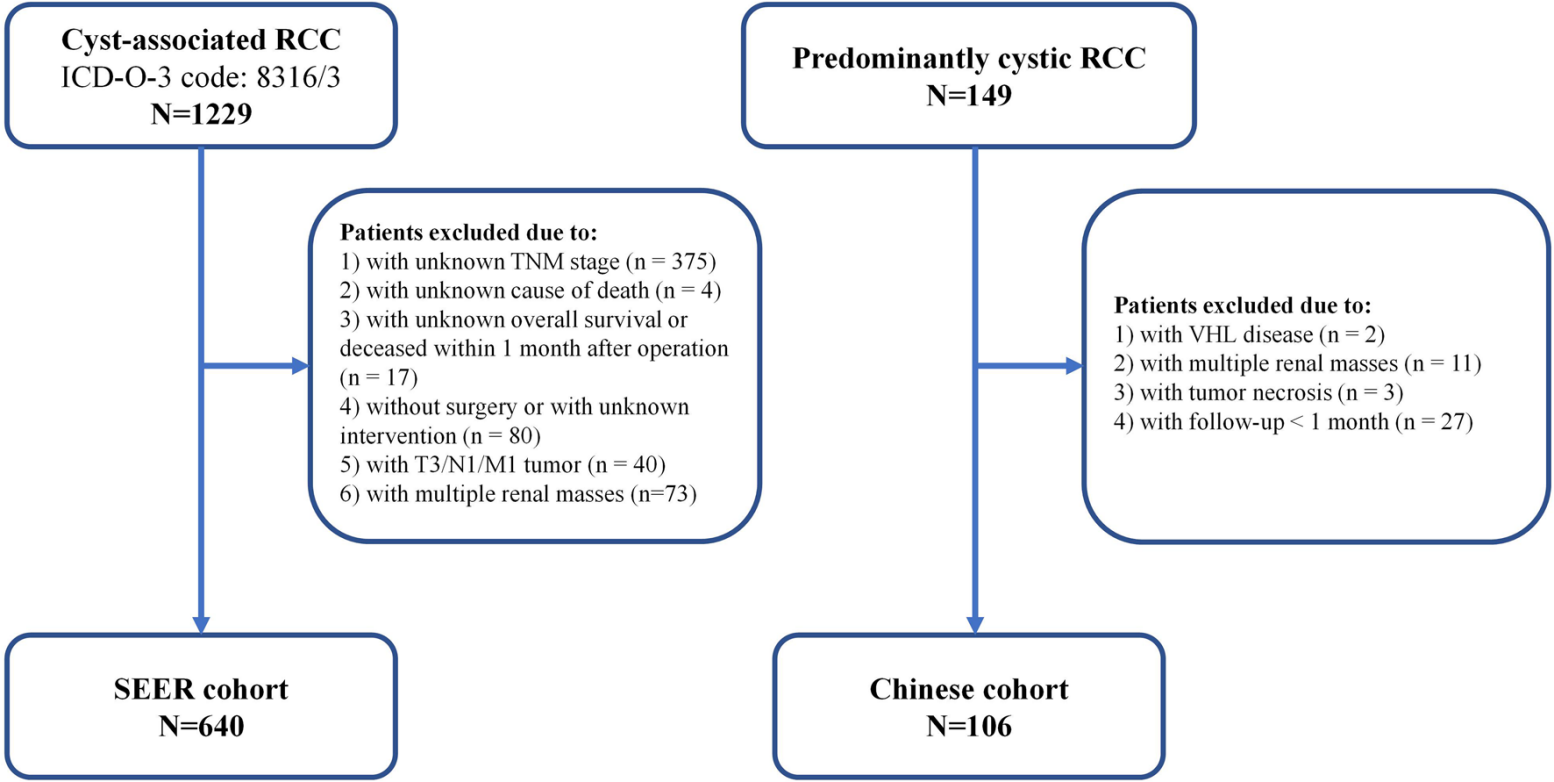
Flowchart illustrating patient selection in this study.

After excluding the patients without the necessary information, a total of 640 patients diagnosed with cyst-associated RCC with a median age of 55 years from the SEER database were screened out. Most of the patients were Caucasian (72.3%) and had T1 cRCC (89.7%). Almost half of the patients (53.8%) underwent RN. Besides, of the 149 patients who underwent RN or PN for cRCC in Shanghai Ruijin Hospital and Shanghai Renji Hospital from 2013 to 2022, 106 patients who met the inclusion criteria were finally included in the analysis (Fig. 1). All the patients were Asian and most of them had T1 cRCC (89.2% in Ruijin and 90.2% in Renji). The median age was 55 years in the Ruijin cohort and 51 years in the Renji cohort. Interestingly, most patients received PN in the Chinese cohort (72.3% in Ruijin and 95.1% in Renji). The patients’ characteristics are shown in Supplementary Table 1.

### Survival analysis for the SEER cohort

**Figure 2:**
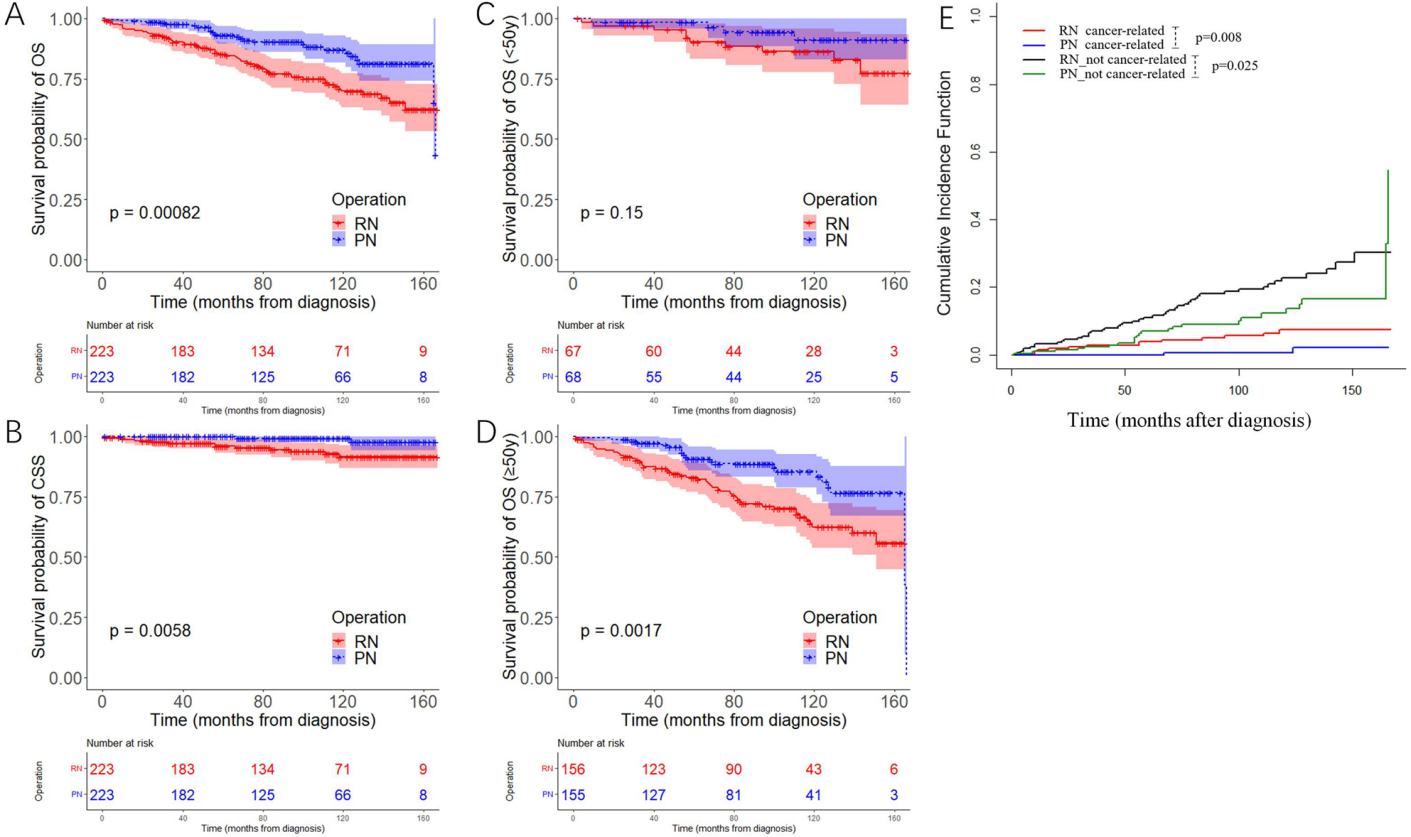
Kaplan–Meier analysis of OS (**A**) and CSS (**B**) in the SEER cohort after PSM. Kaplan–Meier analysis of OS for patients younger (**C**) and older (**D**) than 50y in the SEER cohort after PSM. Competing risk analysis (**E**) in the SEER cohort after PSM. OS: overall survival, CSS: cancer-specific survival, PSM: propensity score-matching.

Kaplan–Meier curves for the SEER cohort before propensity score matching (PSM) suggested that patients who received RN experienced worse cancer-specific survival (CSS) (*p* = 0.018) and overall survival (OS) (*p* < 0.001) compared with those who received PN (Supplementary Fig. 1). However, compared with RN, patients who received PN had a lower level of T stage (95.3% T1 vs. 84.9% T1, *p* < 0.001) and comprised more of Caucasian population (81.8% vs. 64.2%, *p* < 0.001), indicating that these patients may have a better prognosis. The Cox regression analyses showed that age and race were independent risk factors for OS and CSS in the SEER cohort, while PN was significant for OS (hazard ratio [HR] = 0.55, 95% confidence interval [CI] 0.37–0.82, *p* = 0.003) and marginally significant (HR = 0.43, 95%CI 0.18–1.00, *p* = 0.050) for CSS (Supplementary Tables 2, 3). In order to reduce selection bias of the baseline characteristics inherent in each group, a 1:1 PSM analysis was conducted, which included age, race and T stage. The characteristics before and after PSM are listed in Supplementary Table 4. After matching, each group included 223 patients, with similar age (*p* = 0.834), race (*p* = 0.98) and T stage (*p* = 1) in the two groups. Similar to the results before PSM, RN was still associated with worse OS (*p* < 0.001) and CSS (*p* = 0.006) after matching in contrast to PN (Fig. 2A, B). We further analyzed OS in young (< 50 years) and old patients (≥ 50 years) separately, and found that RN did not significantly increase the overall mortality in young patients (*p* = 0.15) unlike in old patients (*p* = 0.002) (Fig. 2C, D). As shown in Fig. 2E, the competing risk model also showed that PN could decrease the cumulative incidences of cancer-related death (*p* = 0.008) and non-cancer-related death (*p* = 0.025).

### Survival and renal function analysis of the Chinese cohort

**Figure 3:**
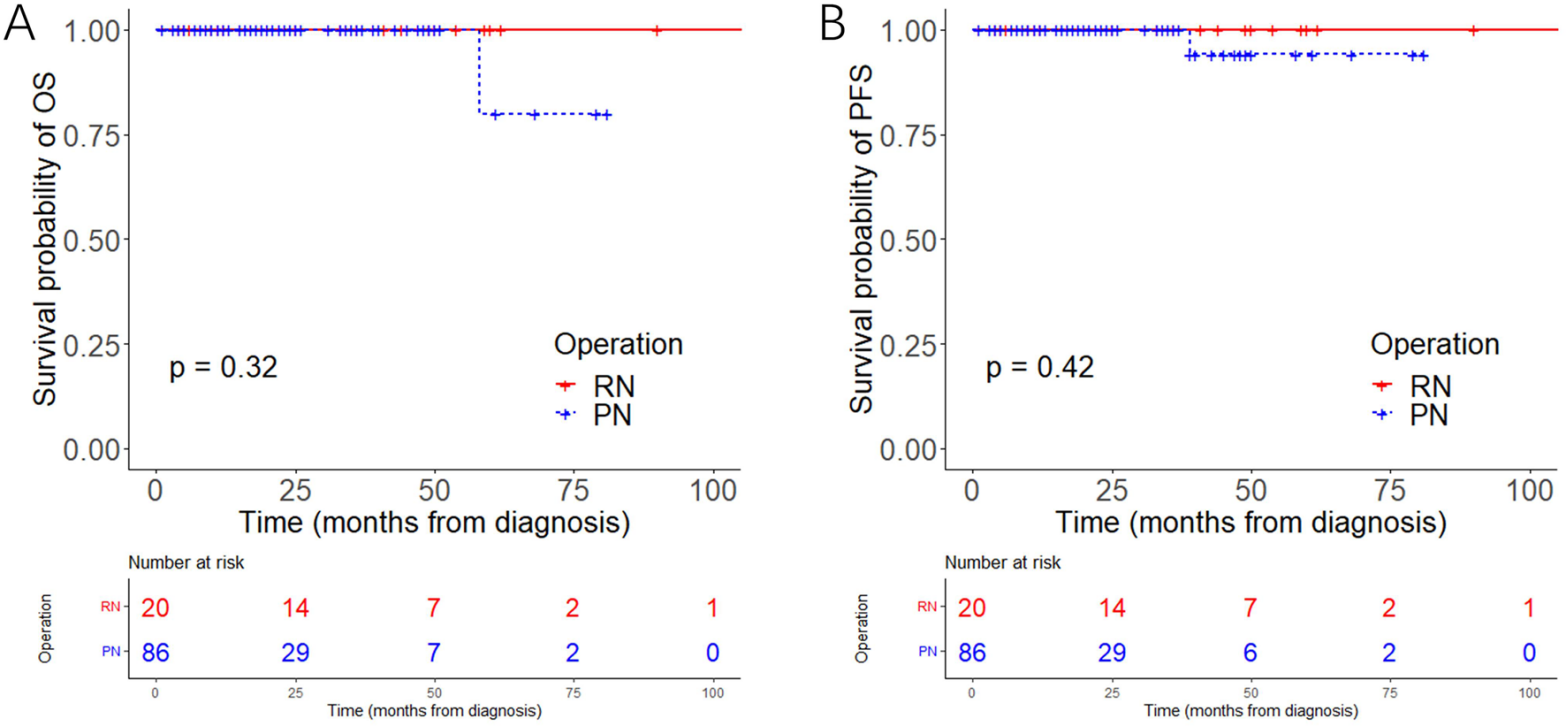
Kaplan–Meier analysis of OS (**A**) and PFS (**B**) in the Chinese cohort. OS: overall survival, PFS: progression-free survival.

**Figure 4:**
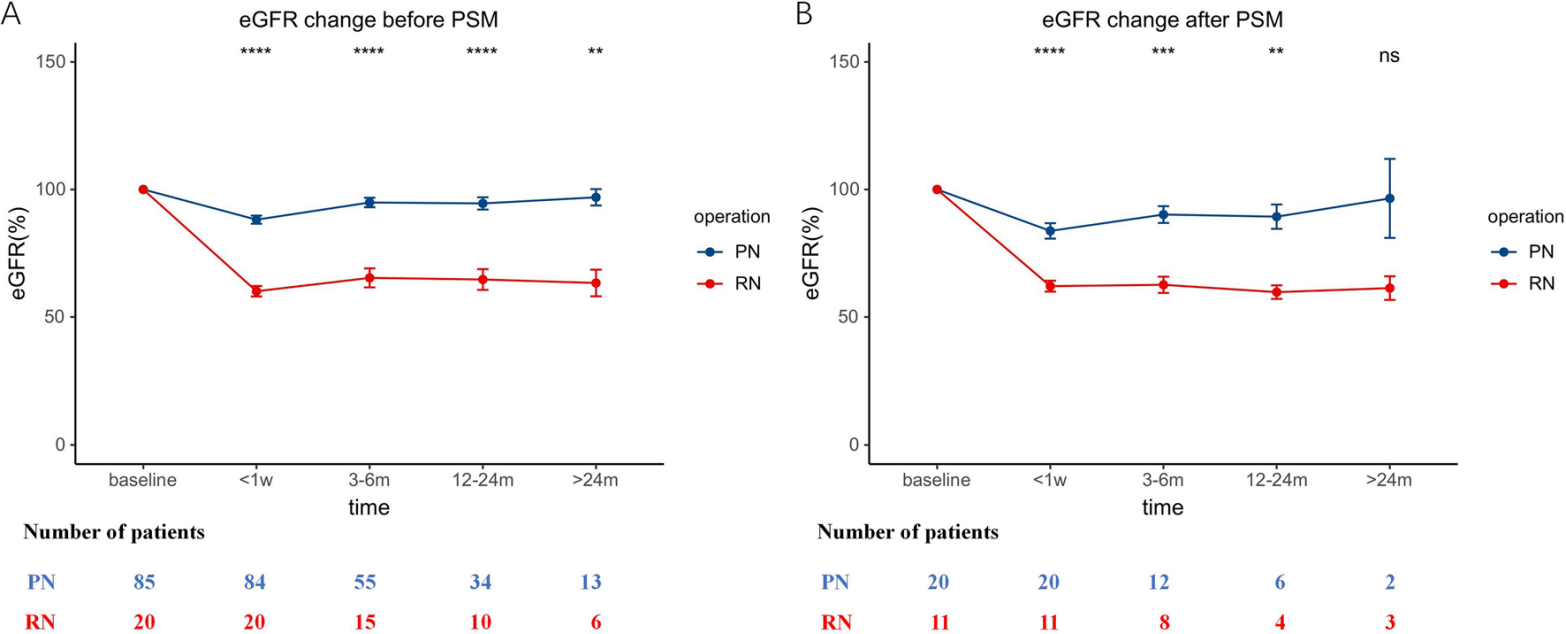
Line chart of eGFR change before (A) and after (B) PSM in the Chinese cohort after operation. ****p < 0.0001, ***p < 0.001, **p < 0.01, *p < 0.05, ns: *p* > 0.05. PSM: propensity score-matching, eGFR: estimated glomerular filtration rate.

No patient died of cRCC in the Chinese cohort, and one died of heart failure in the PN group. In addition, another patient underwent reoperation for tumor recurrence after PN. The Kaplan–Meier analysis showed that PN was as effective as RN for cRCC patients (*p* = 0.32 for OS, *p* = 0.42 for progression-free survival [PFS]) (Fig. 3A, B). As listed in Supplementary Table 5, the estimated blood loss (*p* = 0.644), rate of transfusion (*p* = 0.122) and Clavien-Dindo classification (*p* = 0.172) were similar in the two groups. Most cRCCs in the Chinese cohort were multiocular and recognized as clear cell renal cell carcinoma (ccRCC). No cRCC was with a high WHO/ISUP grade or high expression of Ki67. Next we further explored the change in renal function after RN and PN. Compared with the preoperative data, the mean proportion of estimated glomerular filtration rate (eGFR) preserved after RN was 60.1% within 1 week, 65.3% at 3–6 months, 64.7% at 12–24 months,and 63.3% after 2 years; the mean proportion of eGFR preserved after PN was 87.2% within 1 week, 90.1% at 3–6 months, 90.1% at 12–24 months ,and 96.9% after 2 years. The data showed that PN better preserved the renal function in cRCC patients (Fig. 4A). In the Chinese cohort, patients who underwent PN were younger (*p* = 0.001), their tumor size was generally smaller (*p* < 0.001) and their R.E.N.A.L (for radius, exophytic/endophytic, nearness of tumor to collecting system, anterior/posterior, location relative to polar line) scores were generally lower (*p* < 0.001). Considering that age, hypertension, diabetes, T stage and R.E.N.A.L score may impact on the renal function, we performed PSM to reduce the inherent bias of baseline characteristics in each group. Finally, 11 patients in RN group and 20 patients in PN group were matched. The characteristics before and after PSM are listed in Supplementary Table 6. After PSM, there was no significant difference in the baseline characteristics between the two groups. The mean proportion of eGFR preserved after RN was 62.1% within 1 week, 62.6% at 3–6 months, 61.3% at 12–24 months ,and 62.6% after 2 years; the mean proportion of eGFR preserved after PN was 84.0% within 1 week, 90.0% at 3–6 months, 90.9% at 12–24 months ,and 95.8% after 2 years. The above conclusion was verified based on the results after PSM (Fig. 4B).

## Discussion

cRCC is defined as any malignant renal mass filled with fluid. Four types of cRCC were proposed according to different pathologic mechanisms in the 1980s^9^: (1) unilocular cystic mass; (2) multilocular cystic mass; (3) cystic necrosis; and (4) malignant change of benign cyst. However, the necrosis component in the tumor is always related to an unfavorable prognosis, and some scholars removed it from cRCC^1^. Meanwhile, no definite evidence of a benign cyst undergoing malignant change exists. Therefore, only an unilocular and multilocular cystic malignant renal mass without necrosis is generally defined as cRCC in most recent studies. Multilocular cRCC comprises multiple separate fluid-filled cystic spaces and the stroma between the cysts contains renal cell carcinoma. On the other hand, unilocular cRCC is a fluid-filled mass with a wall covered by tumor cells.

Many urologists opt for RN when resecting cystic renal masses, considering that PN may lead to rupture of the cystic wall, causing tumor cell spread and local recurrence^3,10^. Since the beginning of the twenty-first century, as many studies have demonstrated that cRCC is always non-invasive, more and more surgeons have tried to use PN for cRCC for the sake of preserving the renal function^4,11^. However, whether cyst rupture during PN would increase the risk of recurrence or metastasis remains debatable. A retrospective multicenter study reported that the incidence of cyst rupture was 18.7%, but intraoperative rupture of cRCCs has few oncologic implications^8^. Meanwhile, another retrospective study including 174 patients showed that cyst rupture occurred in 15.5% of their cRCC patients who underwent PN, increasing the risk of tumor recurrence and cancer-specific death^12^. The inconsistent results imply that the impact of cyst rupture during PN remains unclear. Thus, it is necessary to further compare oncologic and other outcomes after PN with outcomes after RN in cRCC patients.

In this study, we screened studies (each study including at least 30 patients) that reported the oncologic outcomes of two surgical methods and were published after 2010^2,13–16^. As shown in Supplementary Table 7, there are limited studies that compared PN with RN in cRCC patients, and the definitions of cRCC are unspecified in some studies. Yet, we did not include studies that only involved multilocular cRCC or MCRNLMP, knowing that no recurrence or metastasis has been reported in MCRNLMP patients before the initiation of the present study^4,5,13,17–19^. Although most previous studies reported that PN was effective as RN, this conclusion needs to be further validated in more cohorts.

In the present study, we first investigated the SEER database, which is the largest series of patients with cRCC. The SEER cohort indicated better OS and CSS in patients who received PN after age, race and T stage adjustment. It should be noted that the difference in OS between PN and RN was only observed in patients older than 50 years, implying that RN may cause death related to advanced age in cRCC patients. Due to the competitive relationship between the death of cRCC and death of other causes, a competing risk analysis was used, revealing that PN could decrease the cumulative incidences of cancer-related death and non-cancer-related death.

Next, we further explored the renal function after operation in the Chinese cohort, as the postoperative renal function might be associated with OS. The line chart demonstrated that PN could better preserve short- and long-term renal function, although the difference was not significant 2 years after the operation in the cohort after PSM because of the limited number of data available. Besides, only one patient experienced oncological progression and only one patient died of heart failure in the Chinese cohort. Due to the lack of oncologic events in the two groups, no Cox regression could be performed, and it was unnecessary to conduct survival analysis again after PSM. The limited scale of the Chinese cohort, especially the RN group, may underestimate the advantage of PN for cRCC, and a multicenter cohort with long-term follow-up is necessary to further confirm our conclusion. However, we think that the priority of PN for cRCC patients can be established as long as there is no evidence showing that PN is worse than RN in survival, as it is universally acknowledged that PN can better preserve the renal function.

There are several limitations in the present study. First, there was no statement regarding the cutoff value of the cystic area for cRCC diagnosis in the SEER cohort. In other words, RCCs with a cystic area < 75% might also be included in the analysis, which may have a poor prognosis. In addition, the follow-up duration was limited in the Chinese cohort. The median follow-up was 21.5 (11–39) months, which might be insufficient to detect recurrence or cancer-specific death for such a non-invasive cancer. Finally, this study was retrospective and 27 patients were lost to follow-up in the Chinese cohort, which may cause selection bias.

## Methods

### Study design

The clinical information of patients diagnosed with cyst-associated RCC between 2000 and 2017 was obtained from the SEER database using SEER*Stat 8.4.0.1 software. The diagnostic code (ICD-O-3 code: 8316/3) was used as the inclusion criteria. The exclusion criteria were patients (1) with unknown TNM stage; (2) with unknown cause of death; (3) with unknown OS or deceased within 1 month after operation; (4) without surgery or with unknown intervention; (5) with T3/N1/M1 tumor; and (6) with multiple renal masses. Multiple renal masses in the SEER cohort were recognized when the patient ID duplicated in a cohort including clear cell renal cell carcinoma (ccRCC), papillary renal cell carcinoma (pRCC), chromo phobe renal cell carcinoma (chRCC) and cRCC from the SEER database. In addition, we investigated 149 consecutive patients pathologically diagnosed with cRCC in Ruijin Hospital (Shanghai, China) and Renji Hospital (Shanghai, China) from 2013 to 2022. In the present study, only tumors with more than a 75% cystic area (as the conventional cutoff value) were included. The exclusion criteria were patients with (1) Von Hippel-Lindau (VHL) disease; (2) multiple renal masses; (3) tumor necrosis; and (4) follow-up periods < 1 month.

### Data collection

Preoperative clinical data included age, gender, body mass index (BMI), the Eastern Cooperative Oncology Group (ECOG) score, the presence of preoperative diabetes mellitus, hypertension, American Society of Anesthesiologists (ASA) classification, tumor side, tumor size, TNM stage and R.E.N.A.L score. All patients underwent computed tomography (CT) scans within 1 month before the operation. The images were reviewed by two urologists and a radiologist who were blinded to the clinicopathological characteristics, and the tumors which visually contained a > 75% cystic area were included in this study. The tumor size was defined as the longest diameter according to the preoperative CT imaging. The R.E.N.A.L score was calculated by two urologists in each institution according to Kutikov’s study^20^.

Postoperative data included estimated blood loss, OS, cancer-specific survival (CSS), progression-free survival (PFS), Clavien-Dindo classification, transfusion, pathology, tumor grade, Ki67, and serum creatinine (SCR) which was detected within 1 week, 3–6 months, 12–24 months and > 24 months after the operation. eGFR was calculated by the Chronic Kidney Disease Collaboration (CKD-EPI) equation proposed in 2009^21^.

### Ethical statement

This study was approved by the ethic committee of each institution (Ruijin Hospital, Shanghai Jiao Tong University School of Medicine, China and Renji Hospital, Shanghai Jiao Tong University School of Medicine, China). This retrospective chart review study involving human participants was in accordance with the ethical standards of the institutional and national research committee and with the 1964 Helsinki Declaration and its later amendments or comparable ethical standards. All patients were informed thoroughly and informed consent was obtained from all patients and also from legal authorized representative of dead patient.

### Statistical analysis

All statistical analyses were conducted using the R software version 4.1.2 (https://www.R-project.org). In the case of categorical variables, frequencies and proportions were presented, while medians and interquartile ranges (IQR) were used to describe continuous variables. The comparison of unordered categorical variables was performed with chi-square test or Fisher exact test. Wilcoxon rank sum test was used for comparing continuous variables and ordered categorical variables. Statistical significance was set at 0.05. In the SEER cohort, a 1:1 nearest-neighbor propensity score matching (PSM) was performed using ‘‘MatchIt’’ R package with baseline characteristics including age, race and T stage. The competing risk analysis was conducted using “cmprsk” package, and the cancer-related death was defined as the main outcome, while death of other causes was defined as the competing outcome. In the Chinese cohort, a 1:2 nearest-neighbor PSM was performed using the following characteristics: age, hypertension, diabetes, T stage and R.E.N.A.L score. The caliper was set as 0.02 standard deviation of the logit of the propensity scores in the SEER cohort, while it was loosened to 0.2 standard deviation of the logit of the propensity scores in the Chinese cohort due to the limited number of the patients. Cox proportional hazards regression analyses, Kaplan–Meier curves and log-rank tests were performed by “survival” R package.

## Conclusion

In the present study, we compared PN with RN in cRCC patients from the SEER database and in an independent cohort in China. The oncological outcome of the PN group was better than the RN group in the SEER cohort. In addition, the Chinese cohort showed that PN could better preserve renal function in cRCC patients, which may contribute to increased OS. We agree that PN is a preferred option for the treatment of cRCC patients. However, as almost all existing studies on cRCC are retrospective, large-scale prospective studies with long follow-up durations are required to further verify the findings and conclusion of the present study.

## Supplementary Information

Supplementary Information.

## Data Availability

The datasets analysed in the current study are available from the corresponding author on reasonable request.
