# Supplementary material for: Comparison of partial nephrectomy and radical nephrectomy for cystic renal cell carcinoma: a SEER-based and retrospective study
Source: Sci Rep. 2023 May 17;13:8052. doi: 10.1038/s41598-023-34950-x (PMC10192435; doi:10.1038/s41598-023-34950-x)
Supplement: Supplementary file 1 — Supplementary Information. [file 41598_2023_34950_MOESM1_ESM.pdf]

Comparison of partial nephrectomy and radical nephrectomy for cystic renal cell carcinoma: A SEER-based and retrospective study

Wenhao Lin[1,+], Zhenggang Yang[2,+], Ling Yan[3,+], Jun Dai[1], Chen Fang[1], Yining Hao[1], Danfeng Xu[1,*], Jin Zhang[2,*], Juping Zhao[1,*]

[1]Department of Urology, Ruijin Hospital, Shanghai Jiao Tong University School of Medicine, Shanghai, China
[2]Department of Urology, Renji Hospital, Shanghai Jiao Tong University School of Medicine, Shanghai, China
[3]Department of Radiology, Ruijin Hospital, Shanghai Jiao Tong University School of Medicine, Shanghai, China

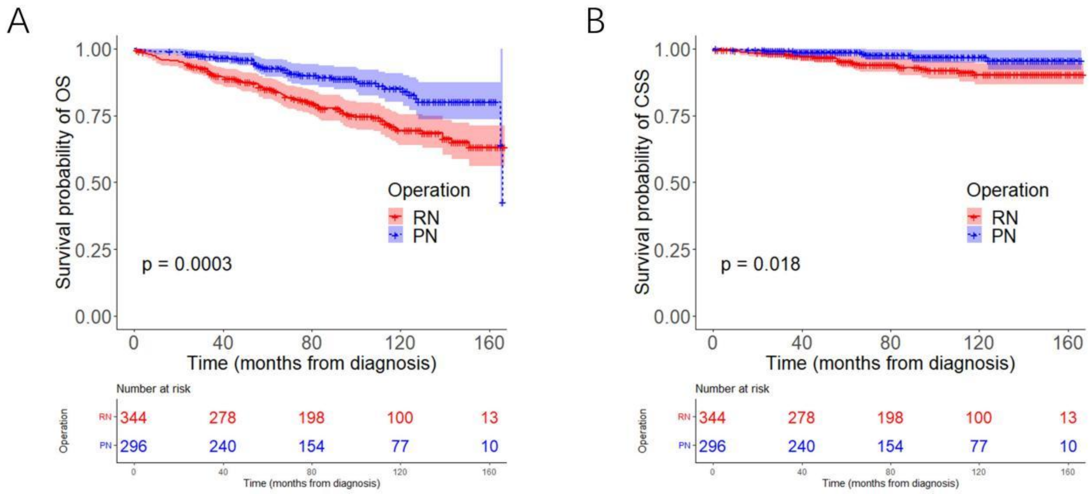

Supplementary Figure 1. Kaplan-Meier analysis of OS (A) and CSS (B) in the SEER cohort before PSM. OS: overall survival, CSS: cancer-specific survival, PSM: propensity score-matching.

Supplementary Table 1. The clinical characteristics of patients in three cohorts.

Variables	SEER cohort (n=640)	Ruijin cohort (n=65)	Renji cohort (n=41)
**Age (median,IQR)**	55 (45-65)	55 (47-62)	51 (39-61)
**Sex (%)**			
**Male**	349 (54.5)	51 (78.5)	25 (61.0)
**Female**	291 (45.5)	14 (21.5)	16 (39.0)
**Race (%)**			
**White**	463 (72.3)	0 (0.0)	0 (0.0)
**Black**	137 (21.4)	0 (0.0)	0 (0.0)
**Other/Unknown**	40 (6.3)	65 (100.0)	41 (100.0)
**T stage (%)**			
**T1**	574 (89.7)	58 (89.2)	37 (90.2)
**T2**	66 (10.3)	7 (10.8)	4 (9.8)
**Intervention (%)**			
**PN**	296 (46.3)	47 (72.3)	39 (95.1)
**RN**	344 (53.8)	18 (27.7)	2 (4.9)

Supplementary Table 2. Univariate and multivariate Cox regression analysis of overall survival in the SEER cohort

Characteristics	Univariate Cox hazard analysis			Multivariate Cox hazard analysis		
	HR	95% CI	P value	HR	95% CI	P value
Age	1.06	1.04-1.08	<0.001	1.06	1.04-1.07	<0.001
Sex						
Female (vs Male)	1.09	0.77-1.56	0.628	—	—	—
Race						
Black (vs White)	2.07	1.42-3.03	<0.001	1.70	1.16-2.49	0.007
Other/Unknown (vs White)	0.66	0.24-1.80	0.414	0.73	0.27-2.01	0.544
T stage						
T2 (vs T1)	0.78	0.42-1.45	0.434	—	—	—
Operation						
PN (vs RN)	0.49	0.33-0.73	<0.001	0.55	0.37-0.82	0.003

HR = hazard ratio.

Supplementary Table 3. Univariate and multivariate Cox regression analysis of cancer specific survival in the SEER cohort

**Characteristics**	**Univariate Cox hazard analysis**			**Multivariate Cox hazard analysis**		
	**HR**	**95% CI**	**P value**	**HR**	**95% CI**	**P value**
**Age**	1.04	1.01-1.07	0.022	1.03	1.00-1.06	0.045
**Sex**						
**Female (vs Male)**	0.93	0.45-1.92	0.853	—	—	—
**Race**						
**Black (vs White)**	2.64	1.27-5.48	0.009	2.18	1.04-4.58	0.039
**Other/Unknown (vs White) ***	—	—	—	—	—	—
**T stage**						
**T2 (vs T1)**	2.01	0.82-4.91	0.128	—	—	—
**Operation**						
**PN (vs RN)**	0.37	0.16-0.87	0.023	0.43	0.18-1.00	0.050

HR = hazard ratio.

*: No patients in this group died of cRCC.

Supplementary Table 4. Baseline parameters in the unmatched and matched groups in the SEER cohort.

Variables	Before PSM			After PSM		
	RN (n=344)	PN (n=296)	P-value	RN (n=223)	PN (n=223)	P-value
**Age (median,IQR)**	55 (45-65)	55 (45-65)	0.195	55 (45-65)	55 (45-65)	0.834
**Sex (%)**			0.885			0.924
**Male**	189 (54.9)	160 (54.1)		119 (53.4)	121 (54.3)	
**Female**	155 (45.1)	136 (45.9)		104 (46.6)	102 (45.7)	
**Race (%)**			<0.001			0.980
**White**	221 (64.2)	242 (81.8)		175 (78.4)	174 (78.0)	
**Black**	101 (29.4)	36 (12.2)		35 (15.7)	35 (15.7)	
**Other/Unknown**	22 (6.4)	18(6.1)		13 (5.8)	14 (6.3)	
**T stage (%)**			<0.001			1
**T1**	292 (84.9)	282 (95.3)		209 (93.7)	209 (93.7)	
**T2**	52 (15.1)	14 (4.7)		14 (6.3)	14 (6.3)	

Supplementary Table 5. Postoperative outcomes before PSM in the Chinese cohort.

Postoperative outcomes		RN (n=20)	PN (n=86)	P-value
**Estimated blood loss (%), ml**				0.644
	**<100**	14 (70.0)	63 (73.3)	
	**100-500**	4 (20.0)	21 (24.4)	
	**>500**	2 (10.0)	2 (2.3)	
**Transfusion (%)**				0.122
	**No**	17 (85.0)	82 (95.3)	
	**Yes**	3 (15.0)	4 (4.7)	
**Clavien-Dindo classification (%)**				0.172
	**I**	17 (85.0)	81 (94.2)	
	**II-III**	3 (15.0)	5 (5.8)	
**Pathology (%)**				0.039
	**ccRCC**	17 (85.0)	83 (96.5)	
	**chRCC**	1 (5.0)	0 (0.0)	
	**pRCC**	1 (5.0)	0 (0.0)	
	**TCRCC**	1 (5.0)	3 (3.5)	
**Multilocular (%)**				0.117
	**No**	0 (0.0)	12 (14.0)	
	**Yes**	20 (100.0)	74 (86.0)	
**WHO/ISUP grade (%)**				0.058
	**1**	4 (20.0)	38 (44.2)	
	**2**	12 (60.0)	35 (40.7)	
	**NA**	4 (20.0)	13 (15.1)	
**Ki67 (%)**				0.371
	**<5%**	9 (45.0)	33 (38.4)	
	**5%-10%**	4 (20.0)	28 (32.6)	
	**NA**	7 (35.0)	25 (29.1)	

ccRCC: clear cell renal cell carcinoma

chRCC: chromophobe renal cell carcinoma

pRCC: papillary renal cell carcinoma

TCRCC: tubulocystic renal cell carcinoma

Supplementary Table 6. The baseline characteristics before and after PSM in the Chinese cohort.

Variables		Before PSM			After PSM	
	RN (n=20)	PN (n=86)	P-value	RN (n=11)	PN (n=20)	P-value
**Age (median,IQR), years**	62 (54-65)	50 (41-59)	0.001	64 (55-66)	61 (50-70)	0.980
**Gender (%)**			0.644			0.705
**Male**	13 (65.0)	63 (73.3)		6 (54.5)	13 (65.0)	
**Female**	7 (35.0)	23 (26.7)		5 (45.5)	7 (35.0)	
**BMI (median,IQR), kg/m²**	24.40 (23.24-26.76)	24.66 (21.82-27.32)	0.611	24.57 (23.16-27.81)	25.44 (22.32-27.94)	0.885
**ECOG (%)**			0.091			0.281
**0**	18 (90.0)	85 (98.8)		9 (81.8)	19 (95.0)	
**1**	2 (10.0)	1 (1.2)		2 (18.2)	1 (5.0)	
**Hypertension (%)**			0.567			1
**No**	13 (65.0)	64 (74.4)		7 (63.6)	13 (65.0)	
**Yes**	7 (35.0)	22 (25.6)		4 (36.4)	7 (35.0)	
**Diabetes (%)**			1			1
**No**	19 (95.0)	79 (91.9)		11 (100.0)	20 (100.0)	
**Yes**	1 (5.0)	7 (8.1)		0 (0.0)	0 (0.0)	
**ASA classification (%)**			0.493			0.406
**I**	13 (65.0)	65 (75.6)		7 (63.6)	16 (80.0)	
**II-III**	7 (35.0)	21 (24.4)		4 (36.4)	4 (20.0)	
**Side (%)**			1			0.262
**Left**	13 (65.0)	54 (62.8)		9 (81.8)	12 (60.0)	
**Right**	7 (35.0)	32 (37.2)		2 (18.2)	8 (40.0)	
**Tumor size (median,IQR), cm**	6.30 (4.45-8.33)	3.20 (2.20-4.50)	<0.001	5.00 (4.05-5.95)	4.50 (3.55-5.33)	0.522
**T stage (%)**			<0.001			1

	**1**	12 (60.0)	83 (96.5)		10 (90.9)	17 (85.0)	
	**2**	8 (40.0)	3 (3.5)		1 (9.1)	3 (15.0)	
**R.E.N.A.L score (%)**				<0.001			0.943
	**4-6**	3 (15.0)	49 (57.0)		3 (27.3)	6 (30.0)	
	**7-9**	7 (35.0)	34 (39.5)		7 (63.6)	12 (60.0)	
	**>9**	10 (50.0)	3 (3.5)		1 (9.1)	2 (10.0)	

Supplementary Table 7. Studies which compared prognosis of cRCC between PN and RN groups.

**Inclusion criteria**	**Cystic area (%)**	**Number of RN**	**Number of PN**	**Median follow up (months)**	**OS**	**CSS**	**PFS**	**Author**
**Histologically proven cystic renal cell carcinomas**	>75	22	22	51[*]	PN=RN[a]	PN=RN[a]	PN=RN[a]	Jhaveri K, 2013
**Histologically proven cystic renal cell carcinomas**	NA	98	70	117	PN=RN[b]	PN=RN[b]	NA	Bhatt J R, 2016
**Cystic renal cell carcinoma**	>75	30	77	58.8	NA	PN=RN[a]	PN=RN[a]	Park J J, 2017
**Renal masses with cystic component on final pathology**	>50	16	122	64.8	NA	PN=RN[a]	PN=RN[a]	Kashan M, 2018
**Histologically proven cystic renal cell carcinomas**	NA	68	84	61	NA	NA	PN=RN[a]	Boissier R, 2019

a: no event happened

b: Cox proportional hazards regression

*: mean follow-up
